# Pregnancy outcomes in women with primary ovarian insufficiency in assisted reproductive technology therapy: a retrospective study

**DOI:** 10.3389/fendo.2024.1343803

**Published:** 2024-04-29

**Authors:** Bo Sun, Lu Li, Yile Zhang, Fang Wang, Yingpu Sun

**Affiliations:** Center for Reproductive Medicine, First Affiliated Hospital of Zhengzhou University, Zhengzhou, China

**Keywords:** primary ovarian insufficiency, pregnancy outcomes, ovarian stimulation, oocyte donation, ART

## Abstract

**Purpose:**

This study aims to retrospectively estimate cumulative reproductive outcomes in women with primary ovarian insufficiency (POI) in assisted reproductive technology (ART) therapy.

**Methods:**

A total of 139 patients diagnosed with POI were reviewed in this study. Firstly, they were divided into two groups according to oocyte origin: using their own oocytes (OG group) or accepting oocyte donations (OD I group). Secondly, the patients were split depending on the pregnancy outcome. In the OG group, nine patients decided to use others’ oocytes after a failure of attempting to use their own, and this population was the oocyte donation II group (OD II group).

**Results:**

There were 88 patients who used their own oocytes, while 51 patients accepted oocyte donations. In the OG group, there are only 10 (7.2%) patients who got pregnant, and patients in the OD group had worse hormone levels (FSH 71.37 ± 4.18 vs. 43.98 ± 2.53, AMH 0.06 ± 0.04 vs. 1.15 ± 0.15, and AFC 0.10 ± 0.06 vs. 1.15 ± 0.15) and more years of infertility (5.04 ± 0.48 vs. 3.82 ± 0.30), which explained why they choose oocyte donation. In all the three groups, baseline characteristics were comparable between pregnant women and non-pregnant women. Of the 10 pregnant patients in the OG group, four of them used luteal-phase short-acting long protocol and had pregnancies successfully in their first cycles.

**Conclusion:**

Ovarian stimulation in POI women requires more cost and time. For those with a stronger desire to have genetic offspring, luteal-phase short-acting long protocol may help them obtain pregnancy rapidly.

## Introduction

Premature ovarian insufficiency (POI) is one of the most common and intractable causes of infertility in women of childbearing age. Due to the reduction of follicle pool or the abnormality of follicle function, patients under 40 years old lose normal ovarian function. The incidence rate of POI in women aged under 40 and 30 is 1% to 2% and 0.1%, respectively (1). The clinical manifestations are menopausal symptoms and long-term sequelae. The long-term sequelae include accelerated cognitive impairment, accelerated cardiovascular aging, infertility, and menopausal symptoms in advance (2). Infertility is the most important one in women during their childbearing years (3). The etiology of POI is diverse and complex, including genetic, autoimmune, metabolic, and iatrogenic, while most patients cannot find a clear cause (4). For women with POI, hormone replacement therapy (HRT) may help to improve the estrogen deficiency symptoms, such as hot flashes, sweating, and osteoporosis, and also prevent cardiovascular diseases. For women with POI with fertility requirements, oocyte donation, for POI patients, is an effective method to make their dream of becoming parents come true, and an increasing number of patients are beginning to receive this treatment (5). However, limiting the number of egg donation and medical ethics restrict this treatment. In recent years, stem cell therapy, *in vitro* activation (IVA), is the new treatment that could be applied (6, 7). Until now, there are several publications that reported a total of 51 patients with POI undergoing IVA therapy. In those studies, 29.4% (15/51) patients with POI had oocyte development, 7.8% (4/51) patients with POI achieved clinical pregnancy, and 5.9% (3/51) patients with POI achieved successful vaginal delivery (6, 8, 9). In clinical trials, there are 29 studies associated with POI but which lack information on pregnancy outcomes. Nevertheless, here are still some issues worth exploring. Since some patients with POI have a very strong wish to obtain a biological child, our study focuses on estimating cumulative pregnancy outcomes in patients with POI in ART therapy.

## Materials and methods

### Selection of patients

**Figure 1:**
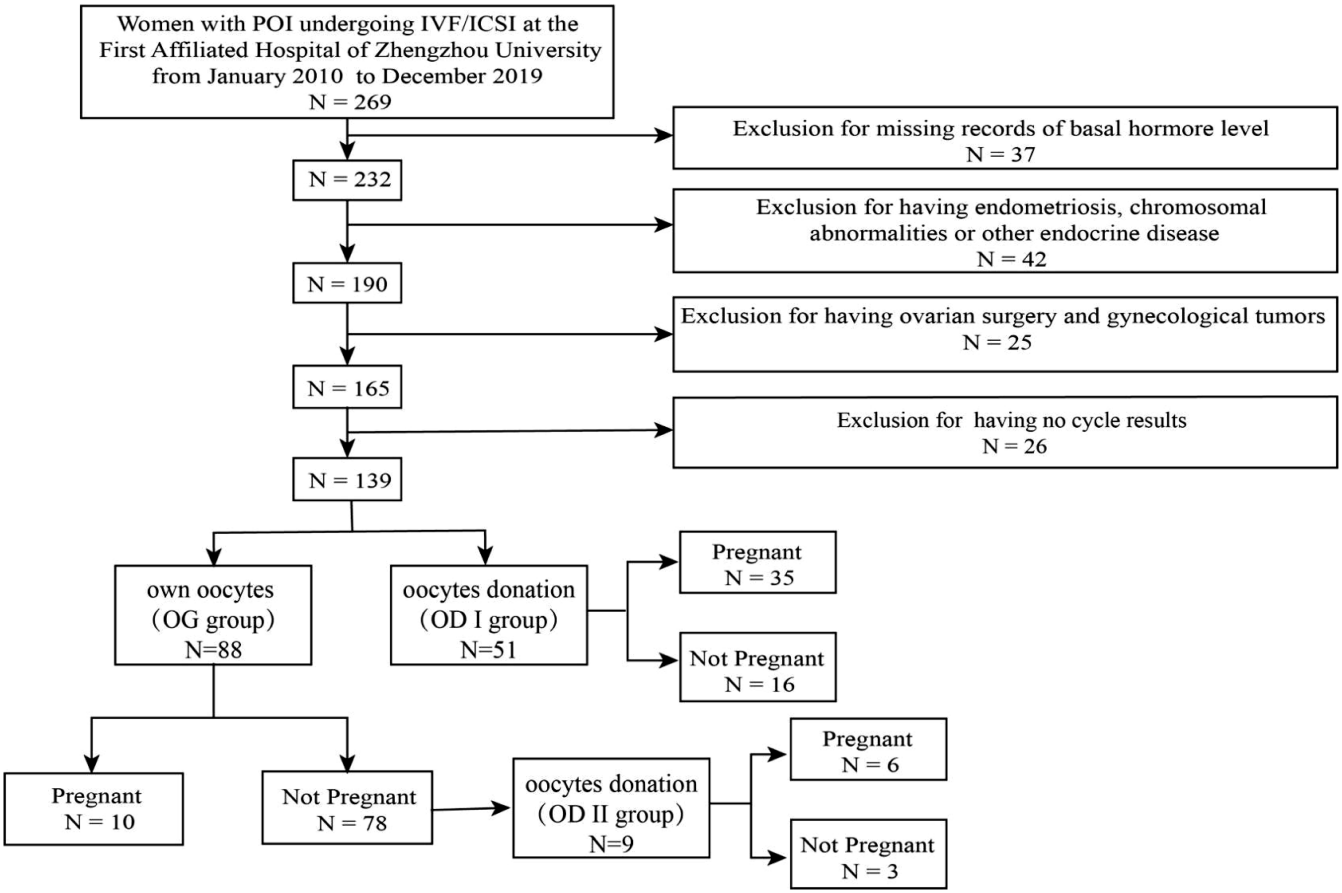
Flowchart of patient screening.

In this retrospective study, a total of 269 women were reviewed. A total of 139 women were included. The participants had undergone ART cycles (including fresh cycles and freeze–thaw cycles) and were diagnosed with POI according to the 2016 ESHRE guidelines, namely: (i) 4 months of persistent oligomenorrhea or amenorrhea and (ii) two tests with follicle-stimulating hormone (FSH) levels above 25 (at an interval of at least 4 weeks) (10). Participants who (1) had endometriosis, chromosomal abnormalities, and other endocrine diseases and (2) had ovarian surgery or gynecological tumors were excluded (**Figure 1**).

### Grouping method

Firstly, the 139 female patients were split into two groups based on oocyte origin: using their own oocytes (OG group) or accepting oocyte donations (OD I group). In the OG group, nine patients decided to use others after a failure of attempting to use their own oocytes, and this population was the oocyte donation II group (OD II group). Each of the three groups was then divided into pregnant or non-pregnant group.

### Ovarian stimulation protocols

#### Follicular-phase long-acting protocol

The patients were injected with 3.75 mg GnRH antagonist (GnRH-a, Tryptorelin, Ferring, Germany) on the 2nd day of menstruation if the ultrasound did not find cysts and follicles >10 mm. The patients will visit the hospital 28 days after the injection to be examined by ultrasound and to check the serum FSH, LH, E2, and progesterone (P) levels. A starting dose of 225–300 IU of human recombinant FSH (rFSH, Gonal F, Serono. Ltd., Switzerland) was administered, with subsequent adjustment of gonadotropin (Gn) use according to follicular growth. The starting dose is according to age, AFC, and basal hormone levels.

#### Luteal-phase short-acting long protocol

Subsequent to the administration of 0.1 mg GnRH antagonist (GnRH-a, Tryptorelin, Ferring, Germany) on day 21 of menstruation, it was used throughout the COH period. rFSH (Gonal F, Serono. Ltd., Switzerland) was given when the patients reached the downregulation criteria. The downregulation criteria were as follows: FSH <5 mIU/mL, LH <5 mIU/mL, E2 <30 pg/mL, and P <0.6 ng/mL; endometrial ≤5 mm; and antral follicle diameter 4–7 mm. Gn was used to induce follicle development. The administration of a starting dose of 225–300 IU of human recombinant FSH (rFSH, Gonal F, Serono. Ltd, Switzerland) lead to a subsequent adjustment of gonadotropin (Gn) use according to follicular growth. The starting dose is according to age, AFC, and basal hormone levels.

#### Mild stimulation protocol

From the 3rd day of menstruation, 2.5 mg letrozole (Furui, Ltd., Jiangsu, China) was used per day, and 300 IU human menopausal gonadotropin (HMG; 75 U/ampoule, Ferring GmbH, Germany) was added 2 days after. When the follicle diameter was >14 mm, monitoring the growth of the follicles was carried out by ultrasound every day.

#### Natural cycle

The follicle monitoring begins around the 6th–8th day of menstruation according to the menstruation cycles. Transvaginal sonography as well as serum sex hormone levels were monitored throughout the whole cycle based on the growth of the follicles.

#### GnRH antagonist protocol

rFSH was administrated on the 2nd day of the cycle, and GnRH antagonist (Cetrorelix, Merck Serono, Europe) was given on the 6th day from the beginning of using rFSH. The follicles were monitored by ultrasound, and serum FSH, LH, E2, and P levels were examined according to the growth of the follicles.

#### Progestin-primed ovarian stimulation

On day 3 of the cycle, medroxyprogesterone acetate (MPA) (Xianju, Ltd., Zhengjiang, China) at 10 mg and HMG 150-225 IU were administered daily. The follicles were monitored after 5 days, and the HMG dose was adjusted depending on the growth of the follicles. The MPA dose was consistent with the trigger day.

In the above-mentioned protocol, ovulation is performed using human chorionic gonadotropin (HCG) (2,000 IU) (Lizhu. Ltd., Guangdong, China) and recombinant HCG (250 μg) (Serono. Ltd., Switzerland) for the follicular-phase long-acting protocol, GnRH antagonist protocol, and luteal-phase short-acting long protocol and using human chorionic gonadotropin (HCG) (10,000 IU) (Lizhu. Ltd., Guangdong, China) for mild stimulation protocol, natural cycle, and progestin-primed ovarian stimulation (PPOS). Egg retrieval was performed 34–36 h later. Depending on the quality of the male partner’s sperm, the fertilization method will be decided.

### Endometrial preparation

Endometrial preparation in all of the patients involved hormone replacement therapy. The detailed endometrial preparation protocol for freeze–thaw cycles has been described in a previous article, including the classification of endometrial types and thickness measurement methods (11). For estrogen–progesterone (EP) cycles, oral estradiol (Progynova, Bayer, Germany) administration began on days 2 to 3 of the target cycle and lasted for about 2 weeks. When the thickness of the endometrium reaches 8 mm and above, the patient is asked to add oil-based progesterone (60 mg). On the same day, the thickness of the endometrium was recorded using transvaginal ultrasound examination. To avoid cavity fluid and other unfavorable conditions, the patients were hospitalized and re-measurement of endometrial thickness was done in the morning of the transplantation day. Luteal supplement was altered to vaginal progesterone gel (90 mg, Crinone 8%; Merck Serono) and oral dydrogesterone (20 mg Duphaston; Abbott) after embryo implantation. Clinical pregnancy was confirmed by ultrasound observation after embryo transfer.

### Embryo cryopreservation and thawing

The cleaved embryos were incubated in an incubator with 6% CO_2_ at 37°C and individually in microdrops (50 μL) containing G1 (Vitrolife) + 5% HSA (Vitrolife). Embryo quality was assessed on day 3 based on the Peter cleavage stage embryo scoring system. Vitrification was used for embryo cryopreservation and thawing.

### Statistical methods

IBM SPSS, 21.0 (IBM Corp., Armonk, NY, USA) was employed. Numerical data were shown as mean ± standard deviation (SD), while categorical variables were shown as % (*n*/*N*). Mann–Whitney test and chi-square test were utilized for continuous and categorical variables, respectively. The threshold was set as two-tailed *P <*0.05.

## Results

### Baseline characteristics and hormone levels of patients with POI undergoing IVF/ICSI

There were 88 (63.3%) patients who used their own oocytes, while 51 (36.7%) patients accepted oocyte donations (**Table 1**). No significant difference was found between the two groups in age (32.73 ± 0.51 vs. 31.27 ± 0.61) and body mass index (BMI) (22.04 ± 0.28 vs. 22.73 ± 0.40). The women in the OG group had lower basal serum FSH (43.98 ± 2.53 vs. 71.37 ± 4.18) and LH (22.45 ± 1.76 vs. 34.53 ± 2.17). The women in the OG group had higher serum E2 (44.24 ± 7.49 vs. 9.97 ± 1.09) and AMH (0.08 ± 0.01 vs. 0.06 ± 0.04). In addition, the women in the OG group had more AFC (1.15 ± 0.15 vs. 0.10 ± 0.06), fewer years of infertility (3.82 ± 0.30 vs. 5.04 ± 0.48), and more numbers of previous pregnancies (0.85 ± 0.14 vs. 0.24 ± 0.06).

**Table 1:** Baseline characteristics and hormone levels of patients with POI undergoing IVF /ICSI.

	OG	OD	P value
Number	88	51	
Age	32.73±0.51	31.27±0.61	0.064
BMI	22.04±0.28	22.73±0.40	0.167
Baseline hormone levels			
FSH (IU/L)	43.98±2.53	71.37±4.18	<0.001
E2(pg/mL)	44.24±7.49	9.97±1.09	<0.001
LH (IU/L)	22.45±1.76	34.53±2.17	<0.001
AMH(ng/ml)	0.08±0.01	0.06±0.04	<0.001
AFC	1.15±0.15	0.10±0.06	<0.001
Type of infertility			0.227
Primary infertility	55	37	
Secondary infertility	33	14	
Years of infertility	3.82±0.30	5.04±0.48	0.031
Number of previous pregnancies	0.85±0.14	0.24±0.06	0.012

Numbers are mean ± standard deviation; BMI, body mass index; FSH, follicle-stimulating hormone; E2, estradiol; LH, luteinizing hormone; AMH, anti-mullerian hormone; AFC, antra follicular count.

### Baseline characteristics and hormone levels of patients in different groups undergoing IVF/ICSI

There were nine patients who accepted oocyte donations after trying using their own oocytes (**Table 2**). In the OG group, the results were comparable between pregnant patients and non-pregnant patients in age (33.40 ± 1.30 vs. 32.64 ± 0.55), BMI (22.58 ± 0.59 vs. 21.98 ± 0.30), and AFC (1.70 ± 0.58 vs. 1.08 ± 0.15), FSH(52.23 ± 11.91 vs. 42.93 ± 2.43, E2 (49.16 ± 26.43 vs. 43.61 ± 7.81), LH (20.00 ± 7.01 vs. 22.76 ± 1.78), and AMH (0.14 ± 0.06 vs. 0.07 ± 0.12). In the OD I group, no statistical difference was found between pregnant women and non-pregnant women in the number of oocyte donations (3.71 ± 0.11 vs. 3.89 ± 0.13), age (31.74 ± 0.73 vs. 30.25 ± 1.07), BMI (22.55 ± 0.48 vs. 23.12 ± 0.74), and AFC (0.09 ± 0.06 vs. 0.13 ± 0.13). Meanwhile, FSH (73.33 ± 5.48 vs. 67.06 ± 5.90), E2 (10.10 ± 1.42 vs. 9.68 ± 1.65), LH (35.15 ± 2.83 vs. 33.16 ± 3.21), and AMH (0.08 ± 0.06 vs. 0.01 ± 0.00) were not significantly different between the two groups. In the OD II group, data were comparable between pregnant patients and non-pregnant patients in the number of oocyte donations (3.83 ± 0.17 vs. 3.33 ± 0.33), age (31.17 ± 1.83 vs. 35.00 ± 2.52), BMI (21.57 ± 1.36 vs. 22.29 ± 2.23), and AFC (0.50 ± 0.22 vs. 0.33 ± 0.33). In the meantime, FSH (92.06 ± 11.95 vs. 50.16 ± 16.82), E2 (9.55 ± 4.672 vs. 18.46 ± 11.62), LH (52.30 ± 5.60 vs. 33.00 ± 11.12), and AMH (0.05 ± 0.03 vs. 0.04 ± 0.03) were not significantly different between the pregnant group and the non-pregnant group.

**Table 2:** Baseline characteristics and hormone levels of patients in different group undergoing IVF /ICSI.

Variables	OG			OD I			OD II		
	pr egnant	non pr egnant	P value	pr egnant	non pr egnant	P value	pr egnant	non pr egnant	P value
Number of patients	10	78		35	16		6	3	
Number of oocytes donation	NA	NA		3.71±0.11	3.89±0.13	0.242	3.83±0.17	3.33±0.33	0.157
Age	33.40±1.30	32.64±0.55	0.678	31.74±0.73	30.25±1.07	0.237	31.17±1.83	35.00±2.52	0.283
BMI	22.58±0.59	21.98±0.30	0.382	22.55±0.48	23.12±0.74	0.556	21.57±1.36	22.29±2.23	0.275
AFC	1.70±0.58	1.08±0.15	0.259	0.09±0.06	0.13±0.13	0.921	0.50±0.22	0.33±0.33	0.655
Baseline hormone levels									
FSH (IU/L)	52.23±11.91	42.93±2.43	0.828	73.33±5.48	67.06±5.90	0.619	92.06±11.95	50.16±16.82	0.110
E2(pg/mL)	49.16±26.43	43.61±7.81	0.874	10.10±1.42	9.68±1.65	0.926	9.55±4.67	18.46±11.62	0.197
LH (IU/L)	20.00±7.01	22.76±1.78	0.086	35.15±2.83	33.16±3.21	0.847	52.30±5.60	33.00±11.12	0.197
AMH (ng/ml)	0.14±0.06	0.07±0.12	0.333	0.08±0.06	0.01±0.00	0.753	0.05±0.03	0.04±0.03	0.423
Type of infertility			1.000			0.942			0.117
Primary infertility	6	49		26	11		6	2	
Secondary infertility	4	29		9	5		0	1	
Duration of infertility	3.90±0.91	3.81±0.31	0.947	5.40±0.56	4.25±0.91	0.094	3.00±0.58	5.00±1.53	0.170
Number of previous pregnancies	1.20±0.57	0.81±0.14	0.709	0.20±0.07	0.31±0.12	0.380	0.00±0.00	0.33±0.33	0.157

Numbers are mean ± standard deviation; BMI, body mass index; FSH, follicle-stimulating hormone; E2, estradiol; LH, luteinizing hormone; AMH, anti-Mullerian hormone; AFC, Antral follicular count.

### Baseline characteristics of pregnant patients in the OG group

There were 10 pregnancies among 88 women in the OG group. The detailed clinical information of each patient is listed in **Table 3**. Five patients were over 35 years old (patients 2, 3, 5, 6, and 10). Only four patients had pregnancies within one cycle (patients 2, 3, 4, and 6). Four pregnancies were obtained after the transfer of fresh embryos (patients 2, 3, 4, and 6) and six pregnancies after a frozen/thawed cycle. Additionally, eight patients achieved live births, including two twin births (patients 2 and 9). The basic serum FSH ranges from 25.37 to 138.4 IU/L, the basic serum E2 ranges from 1.36 to 73.1 pg/mL, the basic serum LH ranges from 6.39 to 68.33 IU/L, and the serum AMH ranges from 0.01 to 0.66 ng/mL. The number of AFC ranges from one to five.

**Table 3:** Baseline characteristics of pregnant patients in OG group.

Serial number	Age	Type of infertility	Total number of cycles	Cycle type*	Protocol	Pregnant outcome	Duration of infertility	BMI	Baseline hormone levels				AFC	Number of previous pregnancy
									FSH (IU/L)	E2 (pg/mL)	LH (IU/L)	AMH (ng/ml)		
1	29	primary infertility	5	ET cycle	E-P protocol	singleton	4	22.9	50.38	32.26	21.97	0.01	0	0
2	36	secondary infertility	1	Fresh cycle	Luteal phase long protocol	twin	8	23.7	32.5	73.1	11.1	0.66	1	1
3	36	primary infertility	1	Fresh cycle	Luteal phase long protocol	singleton	6	21.8	27.73	19.65	7.57	0.31	6	0
4	29	primary infertility	1	Fresh cycle	Luteal phase long protocol	singleton	1	25.7	26.23	14.25	9.09	0.16	3	0
5	36	secondary infertility	4	ET cycle	GnRH-a + E-P protocol	abortion	4	25.5	25.37	21.16	8.38	0.06	0	3
6	36	secondary infertility	1	Fresh cycle	Luteal phase long protocol	singleton	2	21.48	138.4	1.36	68.33	0.01	2	5
7	27	primary infertility	6	ET cycle	E-P protocol	singleton	2	19.9	78.92	28.1	6.44	0.01	0	0
8	32	secondary infertility	5	ET cycle	E-P protocol	singleton	9	21.3	31.19	5	7.89	0.08	2	3
9	33	primary infertility	6	ET cycle	GnRH-a + E-P protocol	twin	2	22	85.56	28.5	52.81	0.01	1	0
10	40	primary infertility	4	ET cycle	Natural cycle	abortion	1	21.5	26.01	15.87	6.39	0.09	2	0

BMI, body mass index; FSH, follicle-stimulating hormone; E2, estradiol; LH, luteinizing hormone; AMH, anti-Mullerian hormone; AFC, antra follicular counts.

*cycle means the cycle when patients get pregnant.

### Ovarian stimulation characteristics of patients with POI undergoing ET cycles

For the 10 pregnancies who used their own oocytes, the ovarian stimulation characteristics of each patient are listed in **Table 4**.

**Table 4:** Ovarian stimulation characteristics of pregnant patients in OG group.

Serial number	Cycle type	Protocol	E2 (pg/mL)	LH (IU/L)	P4 (ng/mL)	Basal endometrial thickness (mm)	Endometrial thickness on HCG (mm)	Length of stimulation n (d)	Total amount of Gn (IU)	Total oocytes retrieved	number of embryos transferred	cycle result
1		PPOS	344.8	1.74	1.3	5	6	3	450	1	/	embryo frozen
		PPOS	267.2	8.13	0.46	5	7	3	450	1	/	Abnormal fertilization
	Fresh cycle	PPOS	362.4	3.37	0.38	5	9	3	450	/	/	No oocytes retrieved
		mild	73.79	13.45	0.45	5	8	9	1725	/	/	No oocytes retrieved
	ET cycle	E-P	123.2	9.72	0.11	5	11	18	64	/	1	pregnant
2	Fresh cycle	short	2260	1.92	0.49	8	12	15	4500	6	2	pregnant
3	Fresh cycle	short	904.9	1.8	0.32	4	8	11	3300	2	2	pregnant
4	Fresh cycle	short	1309	3.44	0.17	4	16	10	3000	5	2	pregnant
		GnRH-a	25.37	24.86	0.07	2	2	7	2100	/	/	Follicle dysplasia
5	Fresh cycle	mild	280.8	1.93	0.26	2	6	13	2250	1	/	embryo frozen
		PPOS	378.9	2.54	0.5	2	5	9	1425	2	/	embryo frozen
	ET cycle	GnRH-a+ E-P	243.6	0.71	0.05	2	7	21	144	/	2	pregnant
6*	Fresh cycle	short	344.8	5.06	0.36	5	9	11	3300	1	1	pregnant
		mild	305.7	8.38	0.68	5	5	1	150	/	/	No oocytes retrieved
7		PPOS	503.6	2.39	0.57	5	3	6	900	1	/	Abnormal fertilization
	Fresh cycle	PPOS	1026	10.88	0.91	5	7	5	750	/	/	No oocytes retrieved
		PPOS	1007	6.6	0.78	5	7	9	1350	2	/	embryo frozen
	ET cycle	E-P	156	69.61	0.64	5	9	9	28	/	/	not transfer
		E-P	184.2	56.99	0.05	5	9	17	78	/	1	pregnant
		mild	231.1	10.59	0.7	6	8	5	750	1	/	embryo frozen
8	Fresh cycle	long	5	14.56	0.41	2	6	5	825	/	/	Follicle dysplasia
		PPOS	128.1	35.5	0.47	5	7	5	750	/	/	No oocytes retrieved
		antagonist	/	/	/	/	/	/	/	/	/	require FET
	ET cycle	E-P	209.2	12.33	0.05	6	8	18	84	/	1	pregnant
		mild	170.3	12.11	0.31	2	8	6	900	1	/	embryo frozen
		mild	313.3	9	0.63	2	6	5	750	/	/	Follicle ovulation in advance
9	Fresh cycle	mild	208.7	27.94	0.58	2	6	3	450	1	/	Abnormal fertilization
		mild	394.1	6.58	0.38	2	7	3	750	1	/	embryo frozen
		mild	558	5.44	0.39	2	5	7	1575	/	/	Follicle ovulation in advance
	ET cycle	GnRH-a+ E-P	197	0.1	0.03	2	10	18	84	/	2	pregnant
10		short	573	5.9	0.69	3	9	19	5700	1	1	not pregnant
	Fresh cycle	mild	192.1	4.2	0.47	3	7	6	900	1	/	embryo frozen
		mild	211.8	4.57	0.42	3	8	7	1050	2	/	embryo frozen
	ET cycle	natural	58.17	12.58	0.73	3	9	5	10	/	2	pregnant

E2, estradiol; P4, progesterone; LH, luteinizing hormone; ET, embryo transfer; on HCG, on the day of human chorionic gonadotropin administration; Gn, gonadotropin ; short, Luteal phase short-acting long protocol; long, Follicular phase long-acting protocol; PPOS, Progestin-primed ovarian stimulation; mild, Mild stimulation protocol; anti, GnRH antagonist protocol. *the patient accepted IVA therapy 156 days ago.

For patient 1, one embryo was frozen in the first PPOS cycle. One oocyte was obtained in the second PPOS cycle, while fertilization was abnormal. In the third PPOS cycle and fourth mild cycle, there were no oocytes retrieved. In the following E-P cycles, the woman got successfully pregnant and delivered.

For patient 2, six oocytes were obtained in the luteal-phase short-acting long protocol. After the transfer of two cleavage embryos, the patient obtained clinical pregnancy and delivered.

For patient 3, luteal-phase short-acting long protocol was administrated, and two oocytes were retrieved and then transferred.

For patient 4, similar to patient 3, she got five oocytes and then transferred two embryos and got pregnant.

For patient 5, ovarian stimulation was made by using three different protocols: GnRH antagonist protocol, mild stimulation protocol, and PPOS protocol. The first cycle was canceled because of follicle dysplasia, while one and two embryos were frozen in the latter two cycles, respectively. Finally, this patient got pregnant after two embryos were implanted in the GnRH-a and E-P cycle.

For patient 6, she got one embryo and got pregnant successfully in the luteal-phase short-acting long protocol after IVA therapy.

For patient 7, no oocyte was obtained in the first mild stimulation cycle. The second PPOS cycle got one oocyte but failed to fertilize, and the third PPOS cycle also got no oocytes. Two embryos were frozen in the fourth PPOS cycle, and she got pregnant after one was transferred in the second E-P cycle.

For patient 8, one embryo was frozen in the first mild stimulation cycle. Unfortunately, there was no mature follicle in the second follicular-phase long-acting protocol cycle, and no oocyte was obtained in the third PPOS cycle. She canceled the fourth cycle and required FET. After transferring one embryo in the following E-P cycle, she got pregnant.

For patient 9, mild stimulation was used; two embryos were frozen in the first and fourth cycles, and the third cycle also got one oocyte. However, normal fertilization failed. Early ovulation was detected in the other two cycles. Finally, this patient got pregnant after two embryos were implanted in the GnRH-a and E-P cycle.

For patient 10, one embryo was transferred in the first luteal-phase short-acting long protocol cycle, but she failed to conceive. The following two mild stimulation cycles obtained three embryos in total, and then two embryos were transferred in the natural cycle and she conceived.

### Ovarian stimulation characteristics of patients with POI undergoing ET cycles

In the patients with POI who had the chance of embryo transfer, we tried to find out the impact factor related to embryo transformation (**Table 5**). In the OG group, the results were comparable between pregnant patients and non-pregnant patients in basal endometrial thickness (3.83 ± 0.70 vs. 4.53 ± 0.48), endometrial thickness on ET day (9.00 ± 0.58 vs. 9.16 ± 0.29), number of embryos transferred (1.50 ± 0.22 vs. 1.42 ± 0.12), and number of good-quality embryos (1.50 ± 0.22 vs. 1.21 ± 0.15). In the OD I group, similar results were obtained between the two groups in basal endometrial thickness (3.81 ± 0.57vs 3.08 ± 0.74), endometrial thickness on ET day (10.71 ± 0.39 vs. 9.50 ± 0.66), number of embryos transferred (1.76 ± 0.07 vs. 1.68 ± 0.11), and number of good-quality embryos (1.35 ± 0.11vs 1.16 ± 0.19). In the OD II group, no statistical difference was found between pregnant patients and non-pregnant patients in basal endometrial thickness (4.50 ± 2.08vs 2.57 ± 1.19), endometrial thickness on ET day (10.00 ± 0.82vs 11.71 ± 0.75), number of embryos transferred (1.83 ± 0.17vs 1 1.57 ± 0.20), and number of good-quality embryos (1.67 ± 0.21 vs. 1.43 ± 0.20).

**Table 5:** Ovarian stimulation characteristics of patients with POI undergoing ET cycles.

ET cycle	OG			OD I			OD II		
	pregnant	not pregnant	P value	pregnant	not pregnant	P value	pregnant	not pregnant	P value
Number	6	16		35	16		6	3	
Number of ART cycles	6	19		35	22		6	7	
Basal endometrial thickness (mm)	3.83±0.70	4.53±0.48	0.436	3.81±0.57	3.08±0.74	0.482	4.50±2.08	2.57±1.19	0.596
Endometrial thickness on ET (mm)	9.00±0.58	9.16±0.29	0.818	10.71±0.39	9.50±0.66	0.503	10.00±0.82	11.71±0.75	0.088
Hormne levels on the day of endometrium transformation									
E2 (pg/mL)	169.23±27.43	384.78±127.56	0.445	157.52±9.24	189.54±15.42	0.054	373.97±187.03	496.35±223.70	0.886
P4 (ng/mL)	0.05±0.01	0.07±0.01	0.767	0.15±0.03	0.11±0.05	0.487	0.40±0.12	0.14±0.04	0.054
LH (IU/L)	15.41±8.62	17.07±4.10	0.567	20.91±2.31	19.63±2.24	0.593	41.80±8.57	23.45±8.53	0.158
Number of embryos transferred	1.50±0.22	1.42±0.12	0.739	1.76±0.07	1.68±0.11	0.528	1.83±0.17	1.57±0.20	0.327
Number of good-quality embryos	1.50±0.22	1.21±0.15	0.330	1.35±0.11	1.16±0.19	0.449	1.67±0.21	1.43±0.20	0.409

Numbers are mean ± standard deviation; E2, estradiol; P4, progesterone; LH, luteinizing hormone; ET, embryo transfer.

### Baseline characteristics and hormone levels of patients with POI with different cycle results

To investigate the factors associated with pregnancy outcomes, we further analyze the baseline characteristics and hormone levels of patients with POI with different cycle results (**Table 6**). The factors that resulted in non-pregnancy included no oocytes retrieved, follicle dysplasia, follicle ovulation in advance, no transferable embryo, and abnormal fertilization. A statistical difference was found between women with no oocytes retrieved and patients with follicle dyspepsia in serum E2 levels on HCG (*p* < 0.001, 323.68 ± 37.33 vs. 34.74 ± 10.85). Patients with follicle dysplasia and patients with follicle ovulation in advance showed a difference in AFC (*p* = 0.011, 0.58 ± 0.15 vs. 2.00 ± 0.72), serum E2 levels on HCG (*p* < 0.001, 34.74 ± 10.85 vs. 354.70 ± 46.72), and serum P4 levels on HCG (*p* = 0.028, 0.39 ± 0.06 vs. 0.66 ± 0.20). Patients with follicle dysplasia and patients with no transferable embryo also showed a difference in serum basal FSH level (*p* = 0.034, 39.23 ± 2.71 vs. 33.05 ± 0.36), serum E2 levels on HCG (*p* < 0.001, 34.74 ± 10.85 vs. 394.51 ± 102.25), and serum P4 levels on HCG (*p* = 0.013, 0.39 ± 0.06 vs. 0.62 ± 0.07). A significant difference was likewise identified between patients with follicle dysplasia and abnormal fertilization in serum E2 levels on HCG (*p* < 0.001, 34.74 ± 10.85 vs. 467.47 ± 97.76) and serum LH levels on HCG (*p* = 0.014, 14.85 ± 2.14 vs. 16.92 ± 6.11).

**Table 6:** Baseline characteristics and hormone levels of patients with POI with different cycle results.

	No oocytes retrieved (1)	P value (2vs1)	Follicle dysplasia (2)	P value (2vs3)	Follicle ovulation in advance (3)	P value (2vs4)	No transferable embryo (4)	P value (2vs5)	Abnormal fertilization (5)
Number	28		26		7		6		8
Number of ART Cycles	31		29		9		7		9
Age	32.39±0.89	0.419	31.42±0.93	0.094	36.14±2.30	0.085	35.00±2.46	0.476	32.88±1.74
BMI	22.33±0.48	0.866	22.45±0.55	0.913	22.33±0.77	0.718	22.88±0.57	0.816	22.21±0.44
Baseline hormone levels									
FSH (IU/L)	46.68±5.33	0.842	39.23±2.71	0.930	42.70±8.06	0.034*	33.05±.36	0.570	46.34±7.84
E2 (pg/mL)	39.20±10.45	0.319	49.64±11.03	0.965	40.58±14.25	0.735	35.04±12.97	0.393	24.33±4.39
LH (IU/L)	21.61±3.09	0.319	24.30±2.66	0.234	19.37±6.54	0.227	15.55±4.20	0.516	22.70±6.55
AMH (ng/ml)	0.07±0.02	0.298	0.04±0.01	0.176	0.08±0.03	0.164	0.14±0.73	0.606	0.09±0.05
AFC	1.14±0.29	0.294	0.58±0.15	0.011*	2.00±0.72	0.101	1.83±0.79	0.174	1.25±0.45
Type of infertility		0.933		0.761		0.152		0.438	
Primary infertility	18		17		5		2		4
Secondary infertility	10		9		2		4		4
Years of infertility Hormone levels on HCG	4.36±0.54	0.155	3.38±0.48	0.607	3.00±1.02	0.941	3.33±1.15	0.168	4.50±0.91
E2 (pg/mL)	323.68±37.33	<0.001***	34.74±10.85	<0.001***	354.70±46.72	<0.001***	394.51±102.25	<0.001***	467.47±97.76
LH (IU/L)	19.23±5.15	0.387	14.85±2.14	0.089	9.33±1.32	0.208	15.98±6.64	0.014*	16.92±6.11
P4 (ng/mL)	0.53±0.08	0.057	0.39±0.06	0.028*	0.66±0.20	0.013*	0.62±0.07	0.548	0.63±0.09

Numbers are mean ± standard deviation; BMI, body mass index; FSH, follicle-stimulating hormone; E2, estradiol; P4, progesterone; LH, luteinizing hormone; AMH, anti-Mullerian hormone; AFC, antra follicular count * P<0.05 ** P<0.01 ***P<0.001.

## Discussion

Since the probability of spontaneous pregnancy in POI patients is only 5%–10%, infertility is a very challenging issue for these patients. The treatment of infertility in patients with POI, who suffer from premature follicular depletion and resistance to exogenous hormones, includes egg donation and IVF/ICSI, the latter of which is often hardly satisfactory (12).

In our study, we first compared the baseline characteristics and hormone levels between patients who used their own oocytes and patients who accepted oocyte donations. It is not difficult to find that the POI patients in the OD group had worse hormone levels and more years of infertility, which made them receive the oocyte donation directly. Next, we compared the baseline characteristics and hormone levels between pregnant patients and non-pregnant patients in the OG, OD I, and OD II groups and sought to identify factors that affect these pregnancy outcomes. However, compared with non-pregnant group, the results in the pregnant group were comparable. Obviously, the rapid treatment for POI with or without some IVF failures is oocyte donation.

In recent years, many researchers have been seeking treatments to stimulate follicle development and elevate the oocyte function of POI patients. The advent of IVA brings a new light to POI patients (9, 13). *In vitro* studies have shown that the activators or inhibitors used in IVA may impair oocyte quality, and this safety uncertainty has implications for the application of IVA (14, 15). Nevertheless, IVA’s use of *in vitro* ovarian cortical fragmentation instead of direct activation of follicles might have a higher efficiency and cause little impairment to the follicles (16). A study by Zhang et al. concluded that 13.75% of women obtained development of follicles after biopsy/scratch, and 1.25% of patients got pregnant and delivered (17). In one autoimmune POI case, *in vitro* maturation (IVM) is used to promote the development of oocytes in the sinus follicular stage into mature eggs, which results in a healthy live birth (18). While IVM is not sufficiently mature, the future holds the promise of maximizing the retention of immature oocytes for these to develop into mature ones for subsequent fertilization and embryo transfer processes.

Although oocyte donation is the better choice and many new treatments occur for POI patients, many of these infertile women are requesting IVF treatment despite the low success rate. In our study, we further analyze the details of pregnant patients in the OG group. For patients with POI undergoing ART, the ultimate goal is to obtain as many mature eggs as possible, although this goal is difficult to achieve and there is no consensus as to which protocol is optimal for this population. Compared to young women with a diminished ovarian reserve, older women with normal ovarian function had a higher embryo implantation rate, but there was no significant difference in live birth rates between the groups, suggesting a discrepancy in egg quality (19). To obtain maximal oocytes and embryos for patients with POI, many studies attempted to explore the optimal ovulation–stimulation protocols. In our study, we performed several protocols—four patients could obtain pregnancy only in the first IVF cycle through the luteal-phase short-acting long protocol and benefited from the better endometrial receptivity of this ovulation–stimulation protocols. However, we had to freeze embryos to transfer in thawed embryo transfer cycle in other ovulation–stimulation protocols.

In the study conducted by Kuang et al., they demonstrated that the PPOS protocol exhibited better suppression of premature LH peaks compared to the antagonist protocol but did not improve the cumulative live birth rate of patients for poor responders (20). We can obtain the same conclusion with this study. Obtaining the maximum number of oocytes through repeated ovarian stimulation and egg retrieval could improve the cumulative live birth rate for POI patients (21). Our study investigated if the patients have a choice to try luteal-phase short-acting long protocol to process fresh IVF cycle transfer, as it will take a shorter time and less cost to achieve pregnancy.

In our study, we also analyzed the ovarian stimulation characteristics of patients with POI in different groups. The results were comparable between the pregnant and non-pregnant patients who had a chance to undergo ET cycles, but we could see that the pregnant ones had more transferred embryos.

Researchers are also exploring various treatments to enhance the pregnancy outcomes of POI patients. In the study of Safak et al., the reproductive outcomes were significantly higher in the random start protocol with clomiphene citrate and gonadotropin treatment than a spontaneous folliculogenesis protocol in women with OPOI (22). Safak et al. also indicated that rescue of the developing oocyte at an early stage of stimulation combined with subsequent embryo transfer can reduce the cycle of retrieval and achieve a favorable outcome for the POI patients (23). Ishizuka et al. analyzed 466 patients with POI undergoing HRT with or without ovarian stimulation (OS), and the follicle growth rate was 48.3% (207/429) per patient in group OS and 5.4% (2/37) in group HRT, while the live birth rate was 5.8% (47/807) in group OS with IVF and no pregnancies occurred in group HRT (24). Moreover, some researchers have also tried using glucocorticoids in combination with high-dose Gn in order to obtain pregnancy in patients with POI (25). A retrospective study comparing the efficacy of glucocorticoids in POI showed that ovulation was achieved in six (20.7%) patients in the dexamethasone group compared to three (10.3%) in the control group, followed by two live births in the dexamethasone group (26). Some studies that attempted to induce ovulation by hHMG stimulation in POI patients and successfully obtained pregnancy were mainly in cases under estrogen replacement (26–28). In the future, we should combine the strengths from different studies to help POI patients obtain pregnancy outcomes in a short time.

Ultimately, we investigated the factors in IVF failures, the largest proportion being no oocytes retrieved and follicle dysplasia. The E2 levels on HCG day in follicle dysplasia is significantly lower than in no oocytes retrieved, follicle ovulation in advance, no transferable embryo, and abnormal fertilization. The AFC is also the least in follicle dysplasia. The reason of follicle dysplasia is linked with POI, and there is no effective solution. Patients with follicle dysplasia should not try more IVF cycles and accept oocyte donation as soon as possible.

Generally, ovarian stimulation in POI women requires more cost and time. For POI patients with a stronger desire to have genetic offspring, luteal-phase short-acting long protocol may help them obtain pregnancy rapidly. If pregnancy cannot be generated within three to four cycles, it is suggested to discourage POI patients from ovarian stimulation and for them to accept oocyte donation.

## Data availability statement

The original contributions presented in the study are included in the article/**Supplementary Material**. Further inquiries can be directed to the corresponding author.

## Ethics statement

The studies involving humans were approved by The Research Ethics Committee of the First Affiliated Hospital of Zhengzhou University. The studies were conducted in accordance with the local legislation and institutional requirements. Written informed consent for participation in this study was provided by the participants’ legal guardians/next of kin.

## Author contributions

BS: Data curation, Supervision, Writing – original draft, Writing – review & editing. LL: Data curation, Software, Writing – original draft. YZ: Conceptualization, Methodology, Visualization, Writing – review & editing. FW: Resources, Software, Visualization, Writing – review & editing. YS: Conceptualization, Investigation, Project administration, Writing – review & editing.
